# Antibiogram, Adhesive Characteristics, and Incidence of Class 1 Integron in *Aeromonas* Species Isolated from Two South African Rivers

**DOI:** 10.1155/2013/127570

**Published:** 2013-09-29

**Authors:** Isoken H. Igbinosa, Vincent N. Chigor, Etinosa O. Igbinosa, Lawrence C. Obi, Anthony I. Okoh

**Affiliations:** Applied and Environmental Microbiology Research Group (AEMREG), Department of Biochemistry and Microbiology, University of Fort Hare, Private Bag X1314, Alice 5700, South Africa

## Abstract

*Aeromonas* species are well distributed in freshwater environments, and their natural susceptibility to antimicrobials renders them interesting candidates for the survey of antimicrobial resistance in freshwater milieu. Water samples were collected from Kat and Tyume rivers in the Eastern Cape province of South Africa, and a total of 45 isolates identified as *Aeromonas* species were recovered from the two rivers. All *Aeromonas* isolates were resistant to oxacillin, penicillin, clindamycin, cephalothin, vancomycin, and rifamycin, while appreciable susceptibilities (89.3 : 94.1%, 82.1 : 94.1%, 85.7 : 88.2%, and 92.9 : 88.2%) were observed against ciprofloxacin, chloramphenicol, nitrofurantoin, and gentamicin from Kat and Tyume rivers, respectively. Multiple antibiotic resistance (MAR) indices ranged from 0.016 to 0.044 for the two rivers. Class 1 integron was detected in about 20% of the isolates, and all the isolates except one showed ability to produce biofilm *in vitro* as weak producers (53.33%), moderate producers (15.56%), and strong producers (28.9%). This investigation provides a baseline data on antibiotic resistance as well as the adhesive characteristics of *Aeromonas* isolates from Tyume and Kat rivers in the Eastern Cape province of South Africa.

## 1. Introduction

*Aeromonas* species are Gram-negative, rod-shaped, non-spore-forming, facultatively anaerobic bacteria that occur ubiquitously and autochthonously in aquatic environments. The *Aeromonas* genus has undergone a number of taxonomic and nomenclature revisions in the past two decades [1]. Initially, *Aeromonas* was placed in the family Vibrionaceae, but successive phylogenetic analyses revealed that *Aeromonas* is not closely related to *Vibrios* and resulted in moving *Aeromonas* to a new family, the Aeromonadaceae [2, 3]. Aeromonads share in common many biochemical characteristics with members of the Enterobacteriaceae; however, they are easily differentiated by being oxidase positive. 

*Aeromonas* species are agents of infection in fish [4] and are associated with human diarrheal diseases and wound infections which may result due to contact with contaminated water [5, 6]. Wound infections may become severe and systemic.* Aeromonas* is also associated with sepsis, respiratory tract, eye and other systemic infections [7]. In fish, *Aeromonas* causes bacterial infections which may pose relatively high resistance to antibiotics including clinically relevant cases and diseases [8]. Treatment of *Aeromonas* infection is usually with the use of antibiotics; however, antimicrobial resistance can make these infections difficult to treat. 

The ubiquity of *Aeromonas* species in aquatic ecosystems and their natural susceptibility to antimicrobials render them interesting candidates for the survey of antimicrobial resistance in freshwater environments [9, 10]. Freshwater streams are usually receptors of many industrial, domestic and agricultural wastes, which could contain antimicrobial agents and antimicrobial-resistant bacteria [10, 11]. Due to diverse microbial population in such ecosystems, freshwater environment provides favourable conditions for the spread of antimicrobial resistance.

Aquatic bacteria such as *Aeromonas* may become reservoirs of antibiotic resistance determinants as a result of influents from diverse sources entering into the river and as such may transfer these antibiotic resistance determinants to other aquatic organisms including pathogenic bacteria [10]. In the United States, a study has shown that several rivers are becoming major reservoirs of antibiotic resistance microorganisms [12]. With widespread commerce and global travel, antibiotic resistant organisms can spread across the globe. The occurrence and distribution of *Aeromonas* in aquatic ecosystems, its emerging significance as a contaminant of water, and the pathogenic potential mediated by mesophilic *Aeromonas* species are all of public health concern [13, 14]. The ability of bacteria to develop multiple-drug resistance result in part from their ability to acquire new antibiotic resistance genes. Mobile elements called integrons determine a site-specific recombination system that is responsible for the acquisition of many antibiotic resistance determinants [15, 16].

Bacterial adherence to surfaces is one of the initial steps leading to biofilm formation and is therefore a significant microbiological event in medicine and the environment [17, 18]. *Aeromonas hydrophila *can attach to and form biofilms on polystyrene, glass surface, stainless steel, and polyvinyl chloride[19]. *Aeromonas* can also attach to solid surfaces and form biofilms in aquatic environments. The presence of *Aeromonas* in biofilm samples from water distribution systems in South Africa has been documented [20]. Biofilm is an irreversible growth of a combination of bacterial micro-colonies on surfaces entrenched in extracellular polysaccharide matrix [21, 22]. The formation of biofilm results in resistance of bacteria to antimicrobial drugs and persistent infections [21] which can lead to the severity of several bacterial diseases affecting both human [23] and animal health [24]. 

Although *Aeromonas* species are well distributed in freshwater habitat and have been assigned as an emerging threat to human health [3, 25], no data exists about the antibiotic susceptibility profiles and adhesive properties of aeromonads from Kat and Tyume rivers in the Eastern Cape province of South Africa. The Food and Agriculture Organization/World Health Organization (FAO/WHO) commission recommends that to prevent waterborne diseases in developing countries, aquatic environments having direct impact on human populations should be characterized physically, chemically, and microbiologically. In view of this recommendation, and as part of our surveillance of reservoir of antibiotic resistant commensal bacteria, this present study aimed to (i) evaluate the levels of antimicrobial resistance in aeromonads isolates from Kat and Tyume rivers, (ii) determine the presence of class 1 and 2 integron associated gene cassette, and (iii) evaluate their biofilm forming capabilities.

## 2. Materials and Methods

### 2.1. Study Area, Sampling and Processing of Samples

 Kat river is located in a semiurban location at geographical coordinates: S32°46.547′ E026°38.456′ while Tyume river is located in a rural community at geographical coordinates: S32°47.279′ E026° 50.520′ in the Eastern Cape province of South Africa. Water samples were collected four times at random between April 2011 and March 2012. Water samples were collected in duplicates using 2 L bottles placed on ice and transported to the laboratory for analysis. Hundred microliter (100 *μ*L) was spread on several Glutamate phenol(GSP) agar (biolab, merck SA); on the other hand, 500 *μ*L was inoculated into 145 mL sterile nutrient broth and incubated in a rotary incubator at 150 rpm overnight at 36°C. At the end of the incubation period, a loopful of the culture was spread and/or streaked on GSP agar, and all agar plates were incubated at 36°C for 24 h. Typical yellow colonies on GSP agar were purified using the same media. Pure colonies were transferred unto nutrient agar plates and subjected to oxidase and catalase tests. Oxidase and catalase positive isolates were further screened for biochemical characteristics using API 20NE kit. The strips were then read, and final identification was made using API lab plus software (bioMerieux, Marcy l'Etoile, France). 

### 2.2. Antimicrobial Susceptibility Testing

Isolates were subcultured on nutrient agar plates incubated for 24 h at 36°C. Colonies were picked from the agar plates, and suspended in normal saline (0.85% w/v), and adjusted to an A_560_ value of 0.12 ± 0.02 (0.5 McFarland standard). The bacterial suspension was spread on the Mueller Hinton agar plates using a sterile swab stick, allowed to dry, and impregnated with antibiotic disk. The antibiotics used were as follows: ciprofloxacin (5 *μ*g), trimethoprim (5 *μ*g), chloramphenicol (3 *μ*g), penicillins (10 *μ*g), clindamycins (2 *μ*g), ofloxacin (5 *μ*g), ampicillin-sulbactam (20 *μ*g), oxacillin (1 *μ*g). ampicillin (25 *μ*g), gentamicin (10 *μ*g), nalidixic acid (30 *μ*g), cefotaxime (30 *μ*g), nitrofurantoin (300 *μ*g), sulfamethoxazole (25 *μ*g), cephalothin (30 *μ*g), erythromycin (15 *μ*g), tetracycline (10 *μ*g), minocycline (30 *μ*g), vancomycin (30 *μ*g), and rifampicin (5 *μ*g). Disks were purchased from Mast Diagnostics (Mast Group Merseyside UK). Plates were incubated at 36°C for 24 h. Diameters of the zones of inhibition were measured and interpreted, as susceptible, intermediate or resistant according to the Clinical Laboratory Standard Guidelines [26]. The frequency of antibiotic-resistant *Aeromonas* isolates was calculated by the following equation: *A*/*B* × 100%, where *A* is the number of isolates resistant to an antibiotic and *B* is the total number of isolates from the sample. The multiple antibiotic resistance (MAR) index of each samples was estimated by the following equation: *a*/(*b* × *c*), where *a* represents the aggregate antibiotic resistance score of all isolates from the sample, *b* represents the number of antibiotics, and *c* represents the number of isolates from the sample as outlined in [27–29].

### 2.3. PCR Detection of Integrons

DNA was extracted following the method described elsewhere [30, 31]. Template DNA was stored at −20°C until it was ready for use. The primer used for the detection of class 1 and class 2 integron is shown in Table 1. The PCR conditions were as follows: initial denaturation at 94°C for 2 min followed by 30 cycles of denaturation at (95°C for 45 s), annealing (56°C for 1 min), extension (72°C for 90 s), and a final extension at 72°C for 10 min.

### 2.4. Biofilm Formation Assay

Quantitatively, biofilm formation among *Aeromonas* isolates was assessed using microtitre plate method described by Stepanovic et al. [32] and Odeyemi et al. [22] with modification. Wells of 96 flat bottomed microtiter plates were filled with 200 *μ*L of Tryptone Soy Broth (TSB) and inoculated with 20 *μ*L of *Aeromonas* isolates grown overnight and standardized to 0.5 McFarland standard. Plates were incubated at 37°C for 24 h. Positive control wells contained *Aeromonas hydrophila* ATCC 7699, and the negative control wells contained uninoculated Tryptone Soy Broth. Contents of each well were aspirated and washed three times with sterile phosphate-buffered saline (PBS). After air-drying, wells were stained with 200 *μ*L of 1% crystal violet for 30 min. The wells were carefully washed with distilled water to remove the excess stain. Plates were allowed to dry at room temperature. Dye bound to adherent cells was resolubilized with 150 *μ*L of absolute ethanol. Microplate reader (Synergy mx Biotek, USA) was used to read the plates at 570 nm wavelength. Average optical density (OD) of each duplicate result was taken including positive and negative controls. Isolates were categorized as nonbiofilm producer (ODi < ODc), weak (ODc < ODi < 0.1), moderate (ODi = 0.1 < 0.12) and strong (ODi > 0.12) producers according to the modified methods of [33, 34]. 

## 3. Statistical Analysis

Susceptibility data were compared by using a Chi-square test with SPSS software for Windows, version 17.0. Both susceptibility and resistance were calculated as percentages with 95% confidence intervals. A *P* value < 0.05 was considered to be statistically significant.

## 4. Results

A total of 45 isolates (17 from Kat river and 28 from Tyume river) were identified as *Aeromonas hydrophila/caviae*. The isolates were evaluated for their antibiotic susceptibilities. Generally, all isolates were resistant to oxacillin, penicillin, clindamycin, cephalothin, vancomycin, and rifamycin, while over 80% of the isolates were susceptible to ciprofloxacin, chloramphenicol, gentamicin, and nitrofurantoin (Table 2). Isolates from both rivers showed a diversified resistance trend to the same antibiotics. For example, all the *Aeromonas* isolates from Tyume river were resistant to ofloxacin, while 89.3% of isolates from Kat river were susceptible to ofloxacin (Table 2). Also, 70.6% of the isolates from Tyume river were resistant against tetracycline, while 82.1% of the isolates from Kat river were susceptible to the antibiotic. Similarly, *Aeromonas* isolates showed 94.1% resistance and 60.7% susceptibility against trimethoprim and 82.3% resistance and 78.6% susceptibility against cefotaxime from Tyume and Kat rivers, respectively, as shown in Table 2. 

**Figure 1:**
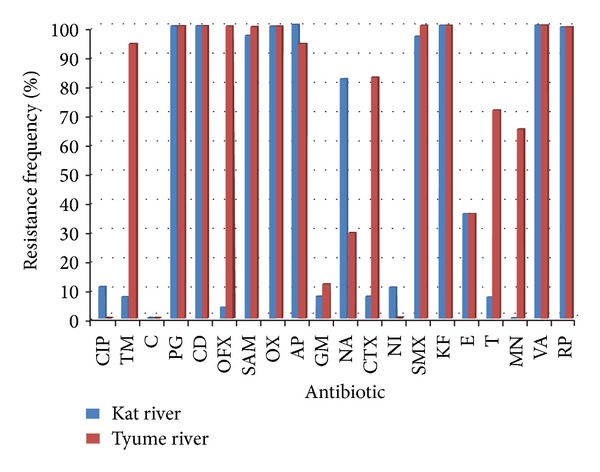
The Frequency of antibiotic-resistant *Aeromonas* isolates from Kat and Tyume rivers. CIP: ciprofloxacin, TM: trimethoprim, C: chloramphenicol, PG: penicillin, CD: clindamycin, OFX: ofloxacin, SAM: ampicillin-sulbactam, OX: oxacillin, AP: ampicillin, GM: gentamicin, NA: nalidixic acid, CTX: cefotaxime, NI: nitrofurantoin, SMX: sulfamethoxazole, KF: cephalothin, E: erythromycin, T: tetracycline, MN: minocycline, VA: vancomycin, RP: rifamycin.

The frequency of antibiotic resistance among the *Aeromonas* isolates from Kat and Tyume rivers is shown in Figure 1. The MAR index of Kat river ranged from 0.026 to 0.044 with a mean value of 0.034. The highest MAR index was observed against only one isolate (MAR 0.044), while six isolates exhibited MAR index of 0.029. *Aeromonas* isolates from Tyume river demonstrated MAR index which ranged from 0.016 to 0.029 with a mean value of 0.0214. Twelve isolates exhibited the lowest MAR index of 0.016. PCR amplification of class 1 and class 2 integron detected class 1 integron in 20% of *Aeromonas* isolates, while class 2 integron was not detected.

**Figure 2:**
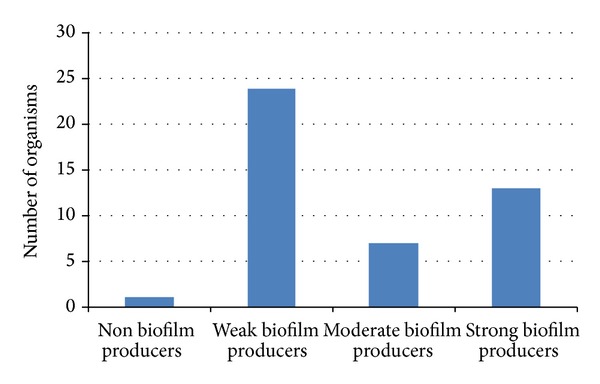
Occurrence of biofilm production of *Aeromonas* isolates in microtitre plates.

*Aeromonas* in this study was able to form biofilms *in vitro *on a polystyrene microtitre plate. Optical densities and biofilm production status of *Aeromonas* isolates from Kat and Tyume rivers are as shown in Table 3. Isolates were categorized into four groups according to the criteria as described above. Weak producers of biofilm which make up 53.33% of the isolates were highest among the groups classified. Thirteen isolates (28.9%) were strong producers while, 7 (15.56%) demonstrated moderate capabilities for biofilm production (Figure 2).  Table 4 shows the adherent characteristics of *Aeromonas* isolates from Tyume and Kat rivers.

## 5. Discussion

The presence of multidrug resistant bacteria in surface water is a major public health burden as drug resistant bacteria could be transferred to humans by means of drinking contaminated water which subsequently contributes to the spread and persistence of antibiotic resistance bacteria in general population and environment [29]. *Aeromonas* species are well distributed in freshwater ecosystems, and their potential to enter distribution systems increases as a result of ineffective water treatment [35]. The result of this investigation demonstrates the presence of multidrug resistant aeromonads in the two rivers assayed. Absolute resistance was observed against several antibiotics with no complete susceptibility to any; however, appreciable susceptibility was observed against ciprofloxacin, chloramphenicol, and nitrofurantoin. Considerable susceptibility was observed for the aminoglycosides tested (gentamicin) with higher susceptibility occurring with isolates from Kat river. Our findings corroborate with the study carried out by Abulhamd [1], which reports susceptibility of *Aeromonas* species isolated from environmental water source to gentamicin. Similar findings of aminoglycosides susceptibility from water sources have been observed [36]. 

Resistance to the penicillin group of antibiotics was observed which can be attributed to *Aeromonas* instinctive resistance to penicillins especially to ampicillin due to the production of *β*-lactamases. Among the cephalosporins tested, 100% resistance was observed against cephalothin, relative susceptibility was observed against cefotaxime with isolates from Kat river, and insignificant susceptibility with *Aeromonas* isolates from Tyume river. Bizani and Brandelli [36] reported 100% resistance of *Aeromonas* species isolated from water distribution system used in a bovine abattoir; however in contrast to our observation, other studies have documented a rare incidence of cephalosporin resistance from *Aeromonas* isolated from water sources [37, 38].

*Aeromonas* isolates from both sources showed similar behaviours to some antibiotics; for instance, isolates from both sources showed high level of susceptibility against chloramphenicol, and none was resistant. Similarly, none of the isolates were susceptible to sulfamethoxazole or cephalothin, while the isolates demonstrated a diversified variation in their susceptibility and resistance against trimethoprim and nalidixic acids. These differences in resistance patterns to antibiotics of the isolates from Kat and Tyume rivers could be as a result of sampling locations (rural and semiurban), anthropogenic activities, and other environmental factors.

Variable susceptibility against the quinolones was observed for *Aeromonas* isolates from both sources, but ofloxacin showed a distinct pattern as it showed 89% susceptibility with isolates from Kat river and absolute resistance against isolates from Tyume river. The reason for these differences may be attributed to enzymatic conduction and selective environmental pressure of *Aeromonas* isolates from these two settings. The presence of multidrug resistant *Aeromonas hydrophila* from aquatic animals including fish [39], eel, and catfish [40, 41] could be as a result of widespread distribution of multidrug resistance among *A. hydrophila *in aquatic (freshwater) habitat; hence, the need for continuous surveillance of emerging and resistance pool of antibiotic resistance determinant.

Integrons are elements that encode a site-specific recombination system that identifies and captures mobile gene cassettes and are closely related to multiple resistances of environmental microorganisms [42]. The role of class 1 integrons in conferring antibiotic resistance to clinical isolates of different bacterial strains is well documented [43–45]. Studies have also shown link between incidence of class 1 integron and antibiotic resistance in bacterial pathogens such as *Escherichia coli* and *Aeromonas salmonicida *[46, 47]. The incidences of integron-bearing *Aeromonas* isolates in our study suggest that these isolates are potential contaminants and that have the possibility for horizontal gene transfer exits. Lukkana et al. [41] has documented the presence of class 1 integron in *Aeromonas hydrophilia* isolated from Nile Tilapia, in Thailand. Rosser and Young [48] documented the incidence of class 1 integrons in 3.6% of bacteria isolated from the Tay estuary, and Lin and Biyela [49] reported the presence of class 1 integron in 58% of *Enterobacteriaceae* isolated from Mhlathuze river in KwaZulu-Natal, Republic of South Africa, and this may imply a wide distribution and persistence of class 1 integron in South African aquatic milieu. The presence of integron in a wide variety of bacteria and in different habitat substantiates the horizontal mobility and stability of this gene capture system [50]. 

Biofilm-producing bacteria have been shown to be associated with numerous human diseases and capable of colonizing a wide range of environments. In aquatic environment, microbial adhesion initiates biofilm formation, exacerbates contamination, reduces the aesthetic quality of the water body, and reduces microbiological safety through augmented survival of pathogens [18, 51]. The result obtained in this study is consistent with the observation of Saidi et al. [52] who found high percentage (95%) of biofilm forming *Aeromonas* species isolated from a river near the seacoast of Monastir, Tunisia. Also, our findings are also similar to the result documented by Odeyemi et al. [22] who found *Aeromonas* isolates from estuary in Malaysia to be biofilm formers with high percentage of weak biofilm producers and strong producers trailing as the second in occurrence. This is the first carried out study on the adhesive properties of environmental *A*. *hydrophila *strains isolated from aquatic source in the Eastern Cape province of South Africa. Ability of *Aeromonas* to form biofilm in aquatic environment enhances the recycling of nutrients and minerals in aquatic environment, thereby promoting the growth of potential pathogen in the aquatic milieu. Motility and flagella play a vital role in adhesion, biofilm formation, and colonization of several pathogenic bacteria, such as *Aeromonas hydrophila *[52, 53]. Our previous study has shown that *Aeromonas* isolates from the studied microhabitat possess some virulent potential [31], and the ability of these virulent strains to form biofilm in aquatic environment may enhance an elevated waterborne dispersal capacity, an attribute that has been related to an elevated risk of bacterial transmission and infectivity [54]. Biofilm formation and development by microorganisms play a vital role in the pathogenesis of a disease; hence, the biofilm forming capability demonstrated *in vitro* by *Aeromonas* isolates from Tyume and Kat rivers further suggests enhanced pathogenic status of these isolates.

## 6. Conclusion

This study provides a baseline data on the antibiotic resistance profile of *Aeromonas* species isolated from Kat and Tyume rivers in the Eastern Cape province of South Africa. The result of this study reveals that the antibiotic resistance patterns of *Aeromonas* species isolated from the two rivers and the incidence of class 1 integron suggest the possibility of horizontal gene transfer of antibiotic resistance determinants in these isolates. The result of this study shows that *Aeromonas* strains from Kat and Tyume rivers have ability to bind to surfaces and form biofilms which is of public health significance as biofilm formation results in resistance of bacteria to conventional antimicrobial agents and persistent infections. 

## Figures and Tables

**Table 1:** Primer set for the detection of class 1 and 2 integron.

Target gene	Primer sequence 5′ → 3′	Size	Reference
Class 1 integron	GGC ATC CAA GCA GCA AG	Variable	[55]
	GGC ATC CAA GCA GCA AG		
Class 2 integron	CGG GAT CCC CGG CAT GCA CGA TTT GTA	Variable	[55]
	GAT GCC ATC GCA AGT ACG AG		

**Table 2:** Antibiogram of *Aeromonas* isolates recovered from Kat river and Tyume river.

Antibiotics	Kat river (%)			Tyume river (%)		
	*S*	*I*	*R*	*S*	*I*	*R*
Ciprofloxacin	89.3	0	10.7	94.1	5.9	0
Trimethoprim	60.7	32.1	7.14	5.9	0	94.1
Chloramphenicol	82.1	17.9	0	94.1	5.9	0
Penicillin	0	0	100	0	0	100
Clindamycin	0	0	100	0	0	100
Ofloxacin	89.3	7.14	3.6	0	0	100
Ampicillin-sulbactam	3.6	0	96.4	0	0	100
Oxacillin	0	0	100	0	0	100
Ampicillin	0	0	100	5.9	0	94.1
Gentamicin	92.9	0	7.14	88.2	0	11.8
Nalidixic acid	14.3	3.6	82.1	70.6	0	29.4
Cefotaxime	78.6	14.3	7.14	11.8	5.9	82.3
Nitrofurantoin	85.7	3.6	10.7	88.2	11.8	0
Sulfamethoxazole	0	3.6	96.4	0	0	100
Cephalothin	0	0	100	0	0	100
Erythromycin	7.14	57.1	35.7	58.8	5.9	35.2
Tetracycline	82.1	21.4	7.14	17.7	11.8	70.6
Minocycline	42.9	57.1	0	23.5	11.8	64.7
Vancomycin	0	0	100	0	0	100
Rifampicin	0	0	100	0	0	100

**Table 3:** Optical densities and biofilm production status of *Aeromonas* species isolated from both Kat and Tyume rivers.

Isolate code	Isolate source (river)	Mean ODi ± SD	Biofilm producing status
Control (TSB)		0.069 ± 0.003	
ISKAE001	Kat	0.084 ± 0.006	Weak
ISKAE002	Kat	0.0725 ± 0.015	Weak
ISKAE003	Kat	0.092 ± 0.021	Weak
ISKAE004	Kat	0.095 ± 0.013	Weak
ISKAE005	Kat	0.102 ± 0.024	Moderate
ISKAE006	Kat	0.073 ± 0.004	Weak
ISKAE007	Kat	0.076 ± 0.037	Weak
ISKAE008	Kat	0.128 ± 0.056	Strong
ISKAE009	Kat	0.123 ± 0.035	Strong
ISKAE010	Kat	0.098 ± 0.009	Weak
ISKAE011	Kat	0.085 ± 0.004	Weak
ISKAE012	Kat	0.119 ± 0.028	Strong
ISKAE020	Kat	0.083 ± 0.016	Weak
ISKWA003	Kat	0.097 ± 0.006	Weak
ISKWA007	Kat	0.073 ± 0.027	Weak
ISKWA013	Kat	0.092 ± 0.045	Weak
ISKWA034	Kat	0.096 ± 0.03	Weak
ISKJA001	Tyume	0.105 ± 0.019	Moderate
ISKJA006	Tyume	0.137 ± 0.018	Strong
ISKJA008	Tyume	0.080 ± 0.009	Weak
ISKJA011	Tyume	0.132 ± 0.04	Strong
ISKJA014	Tyume	0.074 ± 0.015	Weak
ISKJA015	Tyume	0.14 ± 0.002	Strong
ISKJA016	Tyume	0.092 ± 0.021	Weak
ISKJA017	Tyume	0.093 ± 0.005	Weak
ISKJA018	Tyume	0.152 ± 0.007	Strong
ISKJA019	Tyume	0.134 ± 0.012	Strong
ISKJA 021	Tyume	0.142 ± 0.009	Strong
ISKJA 030	Tyume	0.065 ± 0.003	Non-producer
ISKJA 031	Tyume	0.093 ± 0.015	Weak
ISKJA 032	Tyume	0.188 ± 0.002	Strong
ISKJA 033	Tyume	0.163 ± 0.007	Strong
ISKJA 038	Tyume	0.078 ± 0.006	Weak
ISKJA 053	Tyume	0.082 ± 0.007	Weak
ISKJA054	Tyume	0.092 ± 0.004	Weak
ISKJA 055	Tyume	0.119 ± 0.013	Moderate
ISKJA 056	Tyume	0.089 ± 0.002	Weak
ISKJA 062	Tyume	0.098 ± 0.008	Weak
ISKJA 064	Tyume	0.104 ± 0.009	Moderate
ISKWA 033	Tyume	0.12 ± 0.002	Moderate
ISKWA 035	Tyume	0.117 ± 0.011	Moderate
ISKWA 060	Tyume	0.13 ± 0.007	Strong
ISKXA 063	Tyume	0.095 ± 0.004	Weak
ISKXA 074	Tyume	0.149 ± 0.003	Strong
ISKXA 076	Tyume	0.114 ± 0.017	Moderate

**Table 4:** Adherence capability of *Aeromonas* isolates from Kat and Tyume rivers.

River	No. of isolates	Adherence properties			
		NA	WA	MA	SA
Kat	17	0	13	1	3
Tyume	28	1	11	6	10

NA: nonadherent, WA: weakly adherent, MA: moderately adherent, and SA: strongly adherent.
